# Three-dimensional dynamic response of infinite plate resting on multilayered orthotropic foundation under moving loads

**DOI:** 10.1371/journal.pone.0350985

**Published:** 2026-06-12

**Authors:** Chun-li Zhang, Meng Cao, Long Hong, Yun-fei Qi

**Affiliations:** Zhongyuan University of Technology, Zhengzhou, China; China Construction Fourth Engineering Division Corp. Ltd, CHINA

## Abstract

In response to the dynamic response of layered foundation pavement structures caused by traffic loads, a three-dimensional coupled model of an infinite elastic plate on an orthotropic layered foundation under moving loads is established. Based on elastodynamic theory and Kirchhoff thin-plate theory, governing equations are derived by introducing a moving coordinate system and incorporating orthotropic constitutive relations. Through double Fourier transforms and matrix analysis, a single-layer foundation transfer matrix is formulated. The global stiffness matrix is assembled using interlayer continuity conditions, ultimately yielding an analytical solution for the dynamic response of the plate and foundation in the integral transform domain. Combined with ABAQUS finite element simulations, the effects of foundation stratification, soil orthotropy, load parameters, and plate parameters on the dynamic response are systematically studied. The results indicate that the layering of the foundation significantly affects the plate deflection. The orthogonal anisotropy of the first soil layer has a more significant impact on deflection compared to isotropic conditions, and this characteristic needs to be considered in practical engineering. The increase in load speed leads to an increase in peak deflection, while the increase in load frequency results in a decrease in peak deflection. Optimization of plate parameters can effectively suppress deformation, and the amplitude of the deflection curve decreases with the increase of the elastic modulus or thickness of the plate.

## 1. Introduction

The dynamic responses of the foundation and the slab under moving loads have always been a common concern in fields such as traffic engineering and geotechnical engineering. The core of research is to study the complex coupled dynamic responses between the plate and the foundation under dynamic excitations such as vehicle moving loads and impact loads. Yang [1] proposed an integral iterative method for studying the dynamic response of elastic foundation plates under general loads and boundary support conditions using the finite element method and standard finite difference technique. Huang and Thambiratnam [2,3] analyzed the dynamic response of rectangular plates on Winkler foundations under moving concentrated loads and found that the initial velocity and acceleration had a significant impact on the dynamic response of rectangular plates on elastic foundations. Zhang Jianhui [4] and Sheng Y and Huang Y [5] obtained analytical solutions for elastic plates on two-parameter foundations using the superposition method and the element-free Galerkin method. Sun [6] analyzed the influence of moving harmonic line loads and point loads on the displacement of thin plates on Winkler foundations based on dynamic Green’s functions and derived approximate expressions for the critical velocity and critical frequency. Yan Kezhen and Xia Tangdai [7] and Seong Min Kim [8] based on the theory of Winkler foundation elastic thin plates, obtained closed-form solutions for the dynamic response of elastic foundation thin plates under uniform loads and harmonic uniform loads for plates of finite and infinite dimensions, respectively. Patil et al. [9] proposed a finite element solution algorithm that considers the vehicle-pavement interaction. By comparing the responses of finite length and infinite length road surfaces, it was found that under the infinite length road surface model, the pavement surface deflection decreased significantly and the critical speed range narrowed. Savidis SA et al. [10] proposed a hybrid analysis method for dynamic calculation of foundations, greatly contributed to solving the three-dimensional dynamic coupling problem between foundations and elastic plates. Cheng X S [11] investigated the dynamic response of thin plates under moving loads based on the principle of variational methods. Sun L [12] utilized Fourier transform and complex variable function theory to obtain the dynamic response characteristics of plates on viscoelastic foundations under moving loads. Taheri M R [13] developed a novel algorithm based on the finite element method, which fully accounted for the interaction mechanisms between plates under moving loads. Auersch L [14] employed a coupled finite element-boundary element algorithm to derive explicit approximate formulas for infinite plates on a half-space. Hogg A H [15] investigated the dynamic response of an infinite elastic thin plate over a half-space under symmetric loading. Wang Chunling et al. [16,17] solved the bending and dynamic response problems of a four sided free rectangular plate on an elastic half space foundation under arbitrary vertical static loads based on the double Fourier transform. Then, combined with Laplace numerical inverse transform, an analytical solution for a rectangular thin plate on an elastic half space foundation under moving loads was obtained. Wang F et al. [18] developed a solution method based on Fourier transform for the dynamic response problem of anisotropic layered half-space under moving concentrated loads, and obtained a closed-form solution for the three-dimensional dynamic function that satisfies the interlayer continuity condition. Sha Xiangyu et al. [19] established an analytical model for layered soils with fractional viscoelasticity under time-varying loads, and used the complex function method combined with Laplace inverse transform to obtain solutions for stress and displacement responses.

The anisotropy and layered characteristics of soils are widely present in natural sedimentary strata and significantly influence the mechanical response and deformation behavior of foundations. Many scholars have conducted further research. Wang Xiaogang [20] obtained the steady-state response solution under arbitrary harmonic loading through numerical methods and found that the vibration characteristics of plates on transversely isotropic saturated foundations differ significantly from those on traditional isotropic saturated foundations. Ai Z Y et al. [21,22] obtained analytical solutions for transversely isotropic layered foundations using analytical layer element method combined with double Fourier transform. They also studied the response laws of layer characteristics and frequency to the foundation through numerical examples. Yuyan Chen et al. [23] and Zheng BA et al. [24] respectively used Fourier series expansion and inverse Fourier transform combined with stiffness method to obtain the dynamic response solution of transversely isotropic half space soil under moving loads. Muho [25] derived an analytical solution for the dynamic response of a bending elastic plate on a transversely isotropic half space using Fourier series expansion and Frobenius solution method. Feng Duo et al. [26] studied the dynamic response of elastic thin plates on layered foundations and pointed out that moving loads cause asymmetric distribution of vertical displacement amplitudes in the thin plates, and peak values appear in the load zone at critical speeds. Ai Zhiyong et al. [27,28] obtained the dynamic response solutions of rigid circular plates and elastic rectangular plates under uniformly distributed loads based on the analytical layer elements of transversely isotropic layered foundations. Through numerical calculations, they studied the degree of influence of plate soil stiffness ratio, transversely isotropic soil, and layered soil characteristics on the calculation results. Afterwards, combined with the analytical solution of fractional order viscoelastic saturated foundation, a dynamic interaction model of layered isotropic saturated foundation plate was established [29]. Zhang Chunli and Wang Bo et al. [30,31] investigated the dynamic response of an orthogonal anisotropic foundation and an elastic plate in a Cartesian coordinate system. Subsequently, Zhang et al. [32] continued to consider the saturation of soil and established a mechanical model for the interaction between the foundation and an infinite plate under uniformly moving rectangular loads, and derived an analytical solution for the dynamic coupling between an elastic plate and a saturated foundation. Li K. K et al. [33] and Shuai Yang et al. [34] respectively established dynamic governing equations for double-layer and multi-layer isotropic saturated foundations under rectangular moving loads, based on the theory of multiphase porous media and the double Fourier transform, and analyzed the influence mechanisms of load velocity, layered structure, and layer thickness on the dynamic response of the foundations. Zhiqing Zhang et al. [35] constructed a 4 × 4th order efficient formulation for solving dynamic problems in isotropic layered elastic half space based on Cartesian vector functions, and combined with the unconditionally stable bivariate position method to solve dynamic response under time harmonic and moving loads.

However, existing studies primarily focus on simplified isotropic or transversely isotropic foundation models, which cannot adequately capture the complex anisotropic mechanical behavior of natural soils in engineering practice. Systematic investigations into the spatial dynamic response of an orthotropic layered foundation coupled with an infinite elastic plate under moving loads remain notably scarce. In this paper, based on elastic dynamics theory, we integrate the transfer matrix method with integral transform techniques to establish a three-dimensional mechanical model and governing vibration equations for the orthotropic layered foundation–infinite plate coupling system under a uniformly moving rectangular harmonic load, and derive the steady-state closed-form solution. Validated by ABAQUS finite element analysis, systematic parametric studies are conducted to reveal the influence laws of soil stratification, orthotropic parameters, load speed and frequency, and plate stiffness on the plate deflection. The findings are expected to provide theoretical support for the refined design of pavement structures under moving loads.

## 2. Mechanical model and basic governing equations

### 2.1. The mechanical model

**Fig 1:**
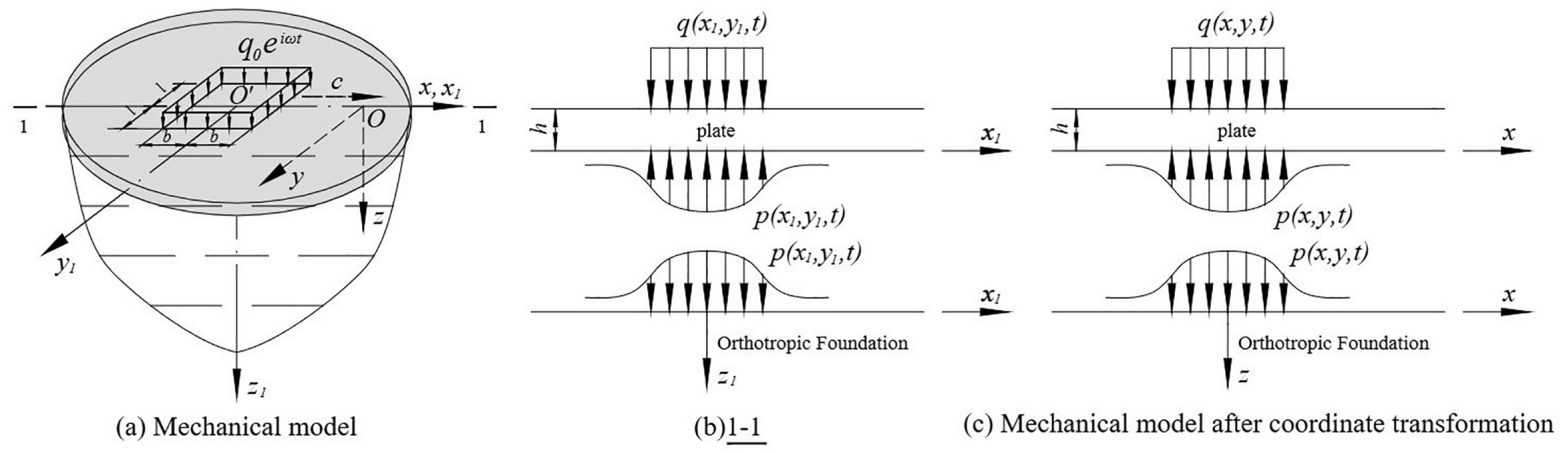
Mechanical model of an infinite plate resting on multilayered orthotropic foundation.


As shown in Fig 1, a mechanical theoretical analysis model of an orthotropic layered foundation and an infinite elastic plate under the rectangular coordinate system is established. Considering the smooth contact between the plate and the foundation, a rectangular harmonic load moving along the positive $x_{\text{1}}$ -axis direction is applied to the thin plate as:

$$\begin{array}{c} q\left(x_{\text{1}},y_{\text{1}},t\right)=\left\{\begin{array}{c} @c@q_{\text{0}}e^{i\omega t}\left|x_{\text{1}}-ct\right|\leq b,\left|y_{\text{1}}\right|\leq l\\ 0\begin{array}{cccc} & & & \begin{array}{cc} & \text{other} \end{array} \end{array} \end{array}\right\} \end{array}$$ (1)

where ω is vibration angular frequency, and c is the load moving velocity.

### 2.2. The basic governing equations

Based on the classical Kirchhoff theory of the elastic thin plate, the governing differential equation of motion for a thin plate under dynamic loads is expressed as:


$$\begin{array}{c} D\nabla^{\text{4}}w_{\text{1}}\left(x_{\text{1}},y_{\text{1}},t\right)+m\frac{\partial^{\text{2}}w_{\text{1}}\left(x_{\text{1}},y_{\text{1}},t\right)}{\partial t^{\text{2}}}=q\left(x_{\text{1}},y_{\text{1}},t\right)-p\left(x_{\text{1}},y_{\text{1}},t\right) \end{array}$$ (2)

where, $D=\frac{Eh^{\text{3}}}{12(1-\mu^{\text{2}})}$ is the flexural rigidity of the elastic plate; E, μ, and h are the elastic modulus, Poisson’s ratio, and thickness of the plate, respectively; $\nabla^{\text{2}}=\left(\frac{\partial^{\text{2}}}{\partial x^{\text{2}}}+\frac{\partial^{\text{2}}}{\partial y^{\text{2}}}\right)$ is the Laplace operator; m is the mass per unit area of the infinite plate; $w_{\text{1}}\left(x_{\text{1}},y_{\text{1}},t\right)$ is the vertical deflection at point $\left(x_{\text{1}},y_{\text{1}}\right)$ and time *t* on the plate overlying the foundation; $p\left(x_{\text{1}},y_{\text{1}},t\right)$ is the vertical reaction force exerted by the foundation on the plate.

Under the moving loads and neglecting the influence of body forces, the three-dimensional dynamic governing equations for an orthotropic anisotropic elastic half-space foundation are expressed as follows:


$$\begin{array}{c} (c_{11}\frac{\partial^{\text{2}}}{\partial {x_{\text{1}}}^{\text{2}}}+c_{66}\frac{\partial^{\text{2}}}{\partial {y_{\text{1}}}^{\text{2}}}+c_{55}\frac{\partial^{\text{2}}}{\partial {z_{\text{1}}}^{\text{2}}})u_{x_{\text{1}}}+(c_{12}+c_{66})\frac{\partial^{\text{2}}u_{y_{\text{1}}}}{\partial x_{\text{1}}\partial y_{\text{1}}}+(c_{13}+c_{55})\frac{\partial^{\text{2}}u_{z_{\text{1}}}}{\partial x_{\text{1}}\partial z_{\text{1}}}=\rho \frac{\partial^{\text{2}}u_{x_{\text{1}}}}{\partial t^{\text{2}}} \end{array}$$ (3a)

$$\begin{array}{c} (c_{66}\frac{\partial^{\text{2}}}{\partial {x_{\text{1}}}^{\text{2}}}+c_{22}\frac{\partial^{\text{2}}}{\partial {y_{\text{1}}}^{\text{2}}}+c_{44}\frac{\partial^{\text{2}}}{\partial {z_{\text{1}}}^{\text{2}}})u_{y_{\text{1}}}+(c_{12}+c_{66})\frac{\partial^{\text{2}}u_{x_{\text{1}}}}{\partial x_{\text{1}}\partial y_{\text{1}}}+(c_{23}+c_{44})\frac{\partial^{\text{2}}u_{z_{\text{1}}}}{\partial y_{\text{1}}\partial z_{\text{1}}}=\rho \frac{\partial^{\text{2}}u_{y_{\text{1}}}}{\partial t^{\text{2}}} \end{array}$$ (3b)

$$\begin{array}{c} (c_{55}\frac{\partial^{\text{2}}}{\partial {x_{\text{1}}}^{\text{2}}}+c_{44}\frac{\partial^{\text{2}}}{\partial {y_{\text{1}}}^{\text{2}}}+c_{33}\frac{\partial^{\text{2}}}{\partial {z_{\text{1}}}^{\text{2}}})u_{z_{\text{1}}}+(c_{13}+c_{55})\frac{\partial^{\text{2}}u_{x_{\text{1}}}}{\partial x_{\text{1}}\partial z_{\text{1}}}+(c_{23}+c_{44})\frac{\partial^{\text{2}}u_{y_{\text{1}}}}{\partial y_{\text{1}}\partial z_{\text{1}}}=\rho \frac{\partial^{\text{2}}u_{z_{\text{1}}}}{\partial t^{\text{2}}} \end{array}$$ (3c)

$$\begin{array}{c} \sigma_{x_{\text{1}}}=c_{11}\frac{\partial u_{x_{\text{1}}}}{\partial x_{\text{1}}}+c_{12}\frac{\partial u_{y_{\text{1}}}}{\partial y_{\text{1}}}+c_{13}\frac{\partial u_{z_{\text{1}}}}{\partial z_{\text{1}}} \end{array}$$ (4a)

$$\begin{array}{c} \sigma_{y_{\text{1}}}=c_{21}\frac{\partial u_{x_{\text{1}}}}{\partial x_{\text{1}}}+c_{22}\frac{\partial u_{y_{\text{1}}}}{\partial y_{\text{1}}}+c_{23}\frac{\partial u_{z_{\text{1}}}}{\partial z_{\text{1}}} \end{array}$$ (4b)

$$\begin{array}{c} \sigma_{z_{\text{1}}}=c_{13}\frac{\partial u_{x_{\text{1}}}}{\partial x_{\text{1}}}+c_{23}\frac{\partial u_{y_{\text{1}}}}{\partial y_{\text{1}}}+c_{33}\frac{\partial u_{z_{\text{1}}}}{\partial z_{\text{1}}} \end{array}$$ (4c)

$$\begin{array}{c} \tau_{y_{\text{1}}z_{\text{1}}}=c_{44}\left(\frac{\partial u_{z_{\text{1}}}}{\partial y_{\text{1}}}+\frac{\partial u_{y_{\text{1}}}}{\partial z_{\text{1}}}\right) \end{array}$$ (4d)

$$\begin{array}{c} \tau_{z_{\text{1}}x_{\text{1}}}=c_{55}\left(\frac{\partial u_{x_{\text{1}}}}{\partial z_{\text{1}}}+\frac{\partial u_{z_{\text{1}}}}{\partial x_{\text{1}}}\right) \end{array}$$ (4e)

$$\begin{array}{c} \tau_{x_{\text{1}}y_{\text{1}}}=c_{66}\left(\frac{\partial u_{y_{\text{1}}}}{\partial x_{\text{1}}}+\frac{\partial u_{x_{\text{1}}}}{\partial y_{\text{1}}}\right) \end{array}$$ (4f)

where, $c_{11}\;c_{66}$ represent the foundation stiffness coefficients; the relationships between the engineering constants $E_{i}$, $\mu_{ij}$, and $G_{ij}$ can be found in reference [36].

As shown in Fig 1, $O^{\prime }x_{\text{1}}y_{\text{1}}z_{\text{1}}$ represents the fixed coordinate system. This study solely addresses the steady-state vibration dynamic response problem under moving loads. To facilitate the analysis, a moving coordinate system Oxyz along the positive direction of the $x_{\text{1}}$ -axis at a velocity *c* is introduced, and a coordinate transformation is performed as:


$$\begin{array}{c} x=x_{\text{1}}-ct,\;y=y_{\text{1}},\;z=z_{\text{1}} \end{array}$$ (5)

By substituting Equation (5) into Equations (2), (3), and (4), respectively, the governing equations are obtained as follows:


$$\begin{array}{c} D_{\text{p}}\nabla^{\text{4}}w\left(x,y\right)+m\left(c^{\text{2}}\frac{\partial^{\text{2}}w\left(x,y\right)}{\partial x^{\text{2}}}-2\text{i}\omega c\frac{\partial w\left(x,y\right)}{\partial x}-\omega^{\text{2}}w\left(x,y\right)\right)=q\left(x,y\right)-p\left(x,y\right) \end{array}$$ (6)

$$\begin{array}{c} \left(c_{11}\frac{\partial^{\text{2}}}{\partial x^{\text{2}}}+c_{66}\frac{\partial^{\text{2}}}{\partial y^{\text{2}}}+c_{55}\frac{\partial^{\text{2}}}{\partial z^{\text{2}}})u_{x}+(c_{12}+c_{66})\frac{\partial^{\text{2}}u_{y}}{\partial x\partial y}+(c_{13}+c_{55})\frac{\partial^{\text{2}}u_{z}}{\partial x\partial z}=\;\rho (c^{\text{2}}\frac{\partial^{\text{2}}u_{x}}{\partial x^{\text{2}}}-2\text{i}\omega c\frac{\partial u_{x}}{\partial x}-\omega^{\text{2}}u_{x}\right) \end{array}$$ (7a)

$$\begin{array}{c} \left(c_{66}\frac{\partial^{\text{2}}}{\partial x^{\text{2}}}+c_{22}\frac{\partial^{\text{2}}}{\partial y^{\text{2}}}+c_{44}\frac{\partial^{\text{2}}}{\partial z^{\text{2}}})u_{y}+(c_{12}+c_{66})\frac{\partial^{\text{2}}u_{x}}{\partial x\partial y}+(c_{23}+c_{44})\frac{\partial^{\text{2}}u_{z}}{\partial y\partial z}=\rho (c^{\text{2}}\frac{\partial^{\text{2}}u_{y}}{\partial x^{\text{2}}}-2\text{i}\omega c\frac{\partial u_{y}}{\partial x}-\omega^{\text{2}}u_{y}\right) \end{array}$$ (7b)

$$\begin{array}{c} \left(c_{55}\frac{\partial^{\text{2}}}{\partial x^{\text{2}}}+c_{44}\frac{\partial^{\text{2}}}{\partial y^{\text{2}}}+c_{33}\frac{\partial^{\text{2}}}{\partial z^{\text{2}}})u_{z}+(c_{13}+c_{55})\frac{\partial^{\text{2}}u_{x}}{\partial x\partial z}+(c_{23}+c_{44})\frac{\partial^{\text{2}}u_{y}}{\partial y\partial z}=\rho (c^{\text{2}}\frac{\partial^{\text{2}}u_{z}}{\partial x^{\text{2}}}-2\text{i}\omega c\frac{\partial u_{z}}{\partial x}-\omega^{\text{2}}u_{z}\right) \end{array}$$ (7c)

$$\begin{array}{c} \sigma_{x}=c_{11}\frac{\partial u_{x}}{\partial x}+c_{12}\frac{\partial u_{y}}{\partial y}+c_{13}\frac{\partial u_{z}}{\partial z} \end{array}$$ (8a)

$$\begin{array}{c} \sigma_{y}=c_{21}\frac{\partial u_{x}}{\partial x}+c_{22}\frac{\partial u_{y}}{\partial y}+c_{23}\frac{\partial u_{z}}{\partial z} \end{array}$$ (8b)

$$\begin{array}{c} \sigma_{z}=c_{13}\frac{\partial u_{x}}{\partial x}+c_{23}\frac{\partial u_{y}}{\partial y}+c_{33}\frac{\partial u_{z}}{\partial z} \end{array}$$ (8c)

$$\begin{array}{c} \tau_{yz}=c_{44}\left(\frac{\partial u_{z}}{\partial y}+\frac{\partial u_{y}}{\partial z}\right) \end{array}$$ (8d)

$$\begin{array}{c} \tau_{zx}=c_{55}\left(\frac{\partial u_{x}}{\partial z}+\frac{\partial u_{z}}{\partial x}\right) \end{array}$$ (8e)

$$\begin{array}{c} \tau_{xy}=c_{66}\left(\frac{\partial u_{y}}{\partial x}+\frac{\partial u_{x}}{\partial y}\right) \end{array}$$ (8f)

where $u_{x}$, $u_{y}$, and $u_{z}$ represent the soil displacements along the *x*, *y*, and *z* directions in the moving coordinate system, respectively; $\sigma_{x}$, $\sigma_{y}$, $\sigma_{z}$, $\tau_{yz}$, $\tau_{zx}$ and $\tau_{xy}$ are the stress components of the soil.

### 2.3. Transfer matrix for single-layer foundation

By coupling Equation (7) with Equation (8) and performing a double Fourier transform on the variables *x* and *y*, the expressions relating the displacements and stresses at the surface of the single-layer orthotropic anisotropic foundation and at any depth *z* are derived as follows:


$$\begin{array}{c} \begin{array}{c} \frac{\partial }{\partial z}\overset{―}{R}\left(\xi ,\eta ,z\right)=A\left(\xi ,\eta \right)\overset{―}{R}\left(\xi ,\eta ,z\right) \end{array} \end{array}$$ (9)

Solving Equation (9) yields the solution of the characteristic equation as follows:


$$\begin{array}{c} \begin{array}{c} \overset{―}{R}\left(\xi ,\eta ,z\right)=e^{zA\left(\xi ,\eta \right)}\overset{―}{R}\left(\xi ,\eta ,0\right) \end{array} \end{array}$$ (10)

Expanding Equation (10) yields


$$\begin{array}{c} \left\{\begin{array}{c} @l@\overset{―}{u_{x}}\left(\xi ,\eta ,z\right)\\ \overset{―}{u_{y}}\left(\xi ,\eta ,z\right)\\ \overset{―}{u_{z}}\left(\xi ,\eta ,z\right)\\ \overset{―}{\sigma_{z}}\left(\xi ,\eta ,z\right)\\ \overset{―}{\tau_{zy}}\left(\xi ,\eta ,z\right)\\ \overset{―}{\tau_{zx}}\left(\xi ,\eta ,z\right) \end{array}\right\}=\left[\begin{array}{cc} @cc@\begin{array}{ccc} T_{11} & T_{12} & T_{13}\\ T_{21} & T_{22} & T_{23}\\ T_{31} & T_{32} & T_{33} \end{array} & \begin{array}{ccc} T_{14} & T_{15} & T_{16}\\ T_{24} & T_{25} & T_{26}\\ T_{34} & T_{35} & T_{36} \end{array}\\ \begin{array}{ccc} T_{41} & T_{42} & T_{43}\\ T_{51} & T_{52} & T_{53}\\ T_{61} & T_{62} & T_{63} \end{array} & \begin{array}{ccc} T_{44} & T_{45} & T_{46}\\ T_{54} & T_{55} & T_{56}\\ T_{64} & T_{65} & T_{66} \end{array} \end{array}\right]\left\{\begin{array}{c} @l@\overset{―}{u_{x}}\left(\xi ,\eta ,0\right)\\ \overset{―}{u_{y}}\left(\xi ,\eta ,0\right)\\ \overset{―}{u_{z}}\left(\xi ,\eta ,0\right)\\ \overset{―}{\sigma_{z}}\left(\xi ,\eta ,0\right)\\ \overset{―}{\tau_{zy}}\left(\xi ,\eta ,0\right)\\ \overset{―}{\tau_{zx}}\left(\xi ,\eta ,0\right) \end{array}\right\} \end{array}$$ (11)

where the specific element expressions of $T_{11}$ …… $T_{66}$ can be found in reference [37].

Transforming Equation (11) into the stiffness matrix form as follows:


$$\begin{array}{c} \left\{\begin{array}{c} @l@\overset{―}{\sigma_{z}}\left(\xi ,\eta ,0\right)\\ \overset{―}{\tau_{zy}}\left(\xi ,\eta ,0\right)\\ \overset{―}{\tau_{zx}}\left(\xi ,\eta ,0\right)\\ \overset{―}{\sigma_{z}}\left(\xi ,\eta ,z\right)\\ \overset{―}{\tau_{zy}}\left(\xi ,\eta ,z\right)\\ \overset{―}{\tau_{zx}}\left(\xi ,\eta ,z\right) \end{array}\right\}=\left[\begin{array}{cc} @cc@K_{\text{1}} & K_{\text{2}}\\ K_{\text{3}} & K_{\text{4}} \end{array}\right]\left\{\begin{array}{c} @l@\overset{―}{u_{x}}\left(\xi ,\eta ,0\right)\\ \overset{―}{u_{y}}\left(\xi ,\eta ,0\right)\\ \overset{―}{u_{z}}\left(\xi ,\eta ,0\right)\\ \overset{―}{u_{x}}\left(\xi ,\eta ,z\right)\\ \overset{―}{u_{y}}\left(\xi ,\eta ,z\right)\\ \overset{―}{u_{z}}\left(\xi ,\eta ,z\right) \end{array}\right\} \end{array}$$ (12)

where $K_{\text{1}}=-{\left[\begin{array}{ccc} @ccc@T_{14} & T_{15} & T_{16}\\ T_{24} & T_{25} & T_{26}\\ T_{34} & T_{35} & T_{36} \end{array}\right]}^{-1}\left[\begin{array}{ccc} @ccc@T_{11} & T_{12} & T_{13}\\ T_{21} & T_{22} & T_{23}\\ T_{31} & T_{32} & T_{33} \end{array}\right]$, $K_{\text{2}}=-{\left[\begin{array}{ccc} @ccc@T_{14} & T_{15} & T_{16}\\ T_{24} & T_{25} & T_{26}\\ T_{34} & T_{35} & T_{36} \end{array}\right]}^{-1}$,


$$\begin{array}{c} K_{\text{3}}=\left[\begin{array}{ccc} @ccc@T_{41} & T_{42} & T_{43}\\ T_{51} & T_{52} & T_{53}\\ T_{61} & T_{62} & T_{63} \end{array}\right]-\left[\begin{array}{ccc} @ccc@T_{44} & T_{45} & T_{46}\\ T_{54} & T_{55} & T_{56}\\ T_{64} & T_{65} & T_{66} \end{array}\right]{\left[\begin{array}{ccc} @ccc@T_{14} & T_{15} & T_{16}\\ T_{24} & T_{25} & T_{26}\\ T_{34} & T_{35} & T_{36} \end{array}\right]}^{-1}\left[\begin{array}{ccc} @ccc@T_{11} & T_{12} & T_{13}\\ T_{21} & T_{22} & T_{23}\\ T_{31} & T_{32} & T_{33} \end{array}\right],\text{\textbackslash{}hspace\{0.17em}\}K_{\text{4}}=\left[\begin{array}{ccc} @ccc@T_{44} & T_{45} & T_{46}\\ T_{54} & T_{55} & T_{56}\\ T_{64} & T_{65} & T_{66} \end{array}\right]{\left[\begin{array}{ccc} @ccc@T_{14} & T_{15} & T_{16}\\ T_{24} & T_{25} & T_{26}\\ T_{34} & T_{35} & T_{36} \end{array}\right]}^{-1}. \end{array}$$

### 2.4. Overall stiffness matrix of layered orthotropic foundation

The continuity conditions of the layered foundation are as follows:


$$\begin{array}{c} \begin{array}{c} {\left\{\begin{array}{c} @c@u_{x}\left(x,y,0\right)\\ u_{y}\left(x,y,0\right)\\ u_{z}\left(x,y,0\right)\\ \sigma_{z}\left(x,y,0\right)\\ \tau_{zy}\left(x,y,0\right)\\ \tau_{zx}\left(x,y,0\right) \end{array}\right\}}_{n}={\left\{\begin{array}{c} @c@u_{x}\left(x,y,h_{n-1}\right)\\ u_{y}\left(x,y,h_{n-1}\right)\\ u_{z}\left(x,y,h_{n-1}\right)\\ \sigma_{z}\left(x,y,h_{n-1}\right)\\ \tau_{zy}\left(x,y,h_{n-1}\right)\\ \tau_{zx}\left(x,y,h_{n-1}\right) \end{array}\right\}}_{n-1} \end{array} \end{array}$$ (13)

By applying Equation (10) to each layer and combining the continuity conditions of the interlayer foundation, the overall stiffness matrix for the entire layered orthotropic system can be assembled as follows:


$$\begin{array}{c} \left\{\begin{array}{c} @c@\overset{―}{\sigma_{z1}}\left(\xi ,\eta ,0\right)\\ \overset{―}{\tau_{zy1}}\left(\xi ,\eta ,0\right)\\ \overset{―}{\tau_{zx1}}\left(\xi ,\eta ,0\right)\\ 0\\ \vdots \\ 0\\ \overset{―}{\sigma_{zN}}\left(\xi ,\eta ,h_{N}\right)\\ \overset{―}{\tau_{zyN}}\left(\xi ,\eta ,h_{N}\right)\\ \overset{―}{\tau_{zxN}}\left(\xi ,\eta ,h_{N}\right) \end{array}\right\}={\left[K\right]}^{e}\left\{\begin{array}{c} @c@\overset{―}{u_{x1}}\left(\xi ,\eta ,0\right)\\ \overset{―}{u_{y1}}\left(\xi ,\eta ,0\right)\\ \overset{―}{u_{z1}}\left(\xi ,\eta ,0\right)\\ \vdots \\ \vdots \\ \vdots \\ \overset{―}{u_{xN}}\left(\xi ,\eta ,h_{N}\right)\\ \overset{―}{u_{yN}}\left(\xi ,\eta ,h_{N}\right)\\ \overset{―}{u_{zN}}\left(\xi ,\eta ,h_{N}\right) \end{array}\right\} \end{array}$$ (14)

where ${\left[K\right]}^{e}=\left[\begin{array}{ccccccccc} @ccccccccc@\overset{\text{1}}{\underset{\text{1}}{K}} & \overset{\text{1}}{\underset{\text{2}}{K}} & & & & & & & \\ \overset{\text{1}}{\underset{\text{3}}{K}} & \overset{\text{1}}{\underset{\text{4}}{K}}+\overset{\text{2}}{\underset{\text{1}}{K}} & \overset{\text{2}}{\underset{\text{2}}{K}} & & & & & & \\ & \overset{\text{2}}{\underset{\text{3}}{K}} & \overset{\text{3}}{\underset{\text{1}}{K}}+\overset{\text{2}}{\underset{\text{4}}{K}} & & & & & & \\ & & & \ddots & & & & & \\ & & & & \ddots & & & & \\ & & & & & \ddots & & & \\ & & & & & & \overset{N-2}{\underset{\text{4}}{K}}+\overset{N-1}{\underset{\text{1}}{K}} & \overset{N-1}{\underset{\text{2}}{K}} & \\ & & & & & & \overset{N-1}{\underset{\text{3}}{K}} & \overset{N-1}{\underset{\text{4}}{K}}+\overset{N}{\underset{\text{1}}{K}} & \overset{N}{\underset{\text{2}}{K}}\\ & & & & & & & \overset{N}{\underset{\text{3}}{K}} & \overset{N}{\underset{\text{4}}{K}} \end{array}\right]$ is the overall stiffness matrix, and $\overset{j}{\underset{i}{K}}$ (i=1,2,3,4, j=1,2⋯N−1,N) is a third-order matrix.

## 3. Solution of the governing equations

### 3.1. Boundary conditions

The surface boundary conditions for the elastic foundation half-space are given as follows:


$$\begin{array}{c} \underset{\left|x\right|\to \infty }{lim}\overset{\left(k\right)}{\underset{x}{u}}\left(x,y,z\right)=0,\text{\textbackslash{}hspace\{0.17em}\}k=0,1,2,3,\cdots N-1,N \end{array}$$ (15a)

$$\begin{array}{c} \underset{\left|y\right|\to \infty }{lim}\overset{\left(k\right)}{\underset{y}{u}}\left(x,y,z\right)=0,\text{\textbackslash{}hspace\{0.17em}\}k=0,1,2,3,\cdots N-1,N \end{array}$$ (15b)

$$\begin{array}{c} \underset{\left|z\right|\to \infty }{lim}\overset{\left(k\right)}{\underset{z}{u}}\left(x,y,z\right)=0,\text{\textbackslash{}hspace\{0.17em}\}k=0,1,2,3,\cdots N-1,N \end{array}$$ (15c)

For a moving harmonic load, all components contain the factor ${\text{e}}^{\text{i}\omega t}$. During the solution process, this factor is initially omitted and then multiplied back into the final results. When the interaction between a layered soil foundation and an elastic plate is considered, the vertical displacements of the plate and the foundation surface are assumed to be continuous, while the friction between the thin plate and the foundation is neglected. Thus, the stress and deformation compatibility boundary conditions are as follows:


$$\begin{array}{c} \sigma_{z}\left(x,y,0,t\right)=-p\left(x,y\right)\left(-b\leq x\leq b,-l\leq y\leq l\right) \end{array}$$ (16a)

$$\begin{array}{c} \tau_{zy}\left(x,y,0,t\right)=0\;\left(-\infty \leq x\leq \infty ,-\infty \leq y\leq \infty \right) \end{array}$$ (16b)

$$\begin{array}{c} \tau_{zx}\left(x,y,0,t\right)=0\;\left(-\infty \leq x\leq \infty ,-\infty \leq y\leq \infty \right) \end{array}$$ (16c)

$$\begin{array}{c} u_{z}\left(x,y,0,t\right)=w\left(x,y,t\right) \end{array}$$ (16d)

Performing the double Fourier transform on equation (16) with respect to *x* and *y*, we can obtain


$$\begin{array}{c} \overset{―}{\sigma_{z}}\left(\xi ,\eta ,0\right)=-\overset{―}{p}\left(\xi ,\eta \right) \end{array}$$ (17a)

$$\begin{array}{c} \overset{―}{\tau_{zy}}\left(\xi ,\eta ,0\right)=0\; \end{array}$$ (17b)

$$\begin{array}{c} \overset{―}{\tau_{zx}}\left(\xi ,\eta ,0\right)=0\; \end{array}$$ (17c)

$$\begin{array}{c} \overset{―}{u_{z}}\left(\xi ,\eta ,0\right)=\overset{―}{w}\left(\xi ,\eta \right) \end{array}$$ (17d)

### 3.2. Solving the equations

By substituting Equations (16) and (17) into Equation (14), and performing matrix manipulations yields:


$$\begin{array}{c} \overset{―}{u_{z}}\left(\xi ,\eta ,0\right)=-f_{31}\overset{―}{p}\left(\xi ,\eta \right) \end{array}$$ (18)

where $f_{31}$ is the element at the third row and first column in the inverse matrix of the overall stiffness matrix $[K{]}^{e}$.

Performing the double Fourier transform with respect to *x* and *y* on the moving harmonic load q(x,y,t) acting on the thin plate yields:


$$\begin{array}{c} \overset{―}{q}\left(\xi ,\eta \right)=\frac{4q_{\text{0}}\;sin\left(\xi b\right)sin\left(\eta l\right)}{\xi \eta } \end{array}$$ (19)

Applying the double Fourier transform on Equation (6) with respect to *x* and *y*, yielding gives:


$$\begin{array}{c} \left[D(\xi^{\text{4}}+2\xi^{\text{2}}\eta^{\text{2}}+\eta^{\text{4}})+m(-c^{\text{2}}\xi^{\text{2}}+2\omega c\xi -\omega^{\text{2}})\right]\overset{―}{w}\left(\xi ,\eta \right)=\overset{―}{q}\left(\xi ,\eta \right)-\overset{―}{p}\left(\xi ,\eta \right) \end{array}$$ (20)

By combining Equations (17), (18), (19) and (20), we can obtain:


$$\begin{array}{c} \overset{―}{p}\left(\xi ,\eta \right)=\frac{\text{1}}{1-\Upsilon }\frac{4q_{\text{0}}\;sin\left(\xi b\right)sin\left(\eta l\right)}{\xi \eta } \end{array}$$ (21)

$$\begin{array}{c} \overset{―}{w}\left(\xi ,\eta \right)=\frac{f_{31}}{\Upsilon -1}\frac{4q_{\text{0}}\;sin\left(\xi b\right)sin\left(\eta l\right)}{\xi \eta } \end{array}$$ (22)

where, $\Upsilon =\left[D(\xi^{\text{4}}+2\xi^{\text{2}}\eta^{\text{2}}+\eta^{\text{4}})+m(-c^{\text{2}}\xi^{\text{2}}+2\omega c\xi -\omega^{\text{2}})\right]f_{31}$

The double Fourier inverse transform of Equation (21) yields the integral-form solution for the contact stress at the interface between the orthotropic anisotropic layered foundation and the elastic plate under moving harmonic loads, as follows:


$$\begin{array}{c} p\left(x,y\right)=\frac{q_{\text{0}}{\text{e}}^{\text{i}\omega t}}{\pi^{\text{2}}}\underset{-\infty }{\int^{+\infty }}\underset{-\infty }{\int^{+\infty }}\frac{\text{1}}{1-\Upsilon }\frac{sin\left(\xi b\right)sin\left(\eta l\right)}{\xi \eta }{\text{e}}^{\text{i}(\xi x+\eta y)}\text{d}\xi \text{d}\eta \end{array}$$ (23)

By implementing the inverse double Fourier transform of Equation (22), the integral-form solution for the deflection of the elastic plate can be expressed as:


$$\begin{array}{c} w\left(x,y\right)=\frac{q_{\text{0}}{\text{e}}^{\text{i}\omega t}}{\pi^{\text{2}}}\underset{-\infty }{\int^{+\infty }}\underset{-\infty }{\int^{+\infty }}\frac{f_{31}}{\Upsilon -1}\frac{sin\left(\xi b\right)sin\left(\eta l\right)}{\xi \eta }{\text{e}}^{\text{i}(\xi x+\eta y)}\text{d}\xi \text{d}\eta \end{array}$$ (24)

## 4. Numerical calculation and analysis of examples

To achieve a certain level of accuracy in the numerical calculations presented in this paper, the intervals −100≤ξ≤100 and −100≤η≤100 are uniformly discretized into 40,960 segments during the discrete fast inverse Fourier transform. The results can satisfy the accuracy requirements.

### 4.1. Parameters of the three-layer system

The pavement structure calculation model takes a three-layer system as an example. The relationships between the soil parameters of each layer are set as $E_{xn}:E_{yn}:E_{zn}=1:1.2:0.8$, $G_{yzn}:G_{zxn}:G_{xyn}=1:1.2:0.8$, and $\mu_{xyn}:\mu_{xzn}:\mu_{yzn}=1:1.2:1.6$. Additionally, $\rho_{\text{1}}=\rho_{\text{2}}=\rho_{\text{3}}$ and $\mu_{xy1}=\mu_{xy2}=\mu_{xy3}$. The remaining parameters are provided in Tables 1–4.

**Table 1:** Soil parameters.

Soil Parameters	$\mu_{xyn}$	$\rho_{n}/{(kg/m}^{3})$	$\Delta h_{n}/\left(𝐦\right)$
**first layer**	0.25	1800	2
**second layer**	0.25	1800	2
**third layer**	0.25	1800	infinite

**Table 2:** Parameters of the Plate.

Parameters	E/(Pa)	μ	$\rho_{0}/{(kg/m}^{3})$	h/(𝐦)
**Numerical Values**	3.0 × 10^10^	0.15	2400	0.25

**Table 3:** Loading parameters.

Parameters	$\begin{array}{c} b/\\ \left(𝐦\right) \end{array}$	$\begin{array}{c} l/\\ \left(𝐦\right) \end{array}$	$\begin{array}{c} q_{0}/\\ \left(kPa\right) \end{array}$	$\begin{array}{c} c/\\ \left(m/s\right) \end{array}$	$\begin{array}{c} f/\\ \left(Hz\right) \end{array}$
**Numerical Values**	1.5	0.75	100	35	8

**Table 4:** Calculation case parameters.

Case	First Layer		Second Layer		Third Layer	
	$\begin{array}{c} E_{x1}/\\ \left(MPa\right) \end{array}$	$\begin{array}{c} G_{yz1}/\\ \left(MPa\right) \end{array}$	$\begin{array}{c} E_{x2}/\\ \left(MPa\right) \end{array}$	$\begin{array}{c} G_{yz2}/\\ \left(MPa\right) \end{array}$	$\begin{array}{c} E_{x3}/\\ \left(MPa\right) \end{array}$	$\begin{array}{c} G_{yz3}/\\ \left(MPa\right) \end{array}$
**1**	50	20	50	20	50	20
**2**	50	20	100	40	25	10
**3**	50	20	25	10	100	40
**4**	100	40	50	20	25	10
**5**	25	10	50	20	100	40
**6**	100	40	25	10	50	20
**7**	25	10	100	40	50	20

### 4.2. Comparison of analytic solutions with the literature results

**Fig 2:**
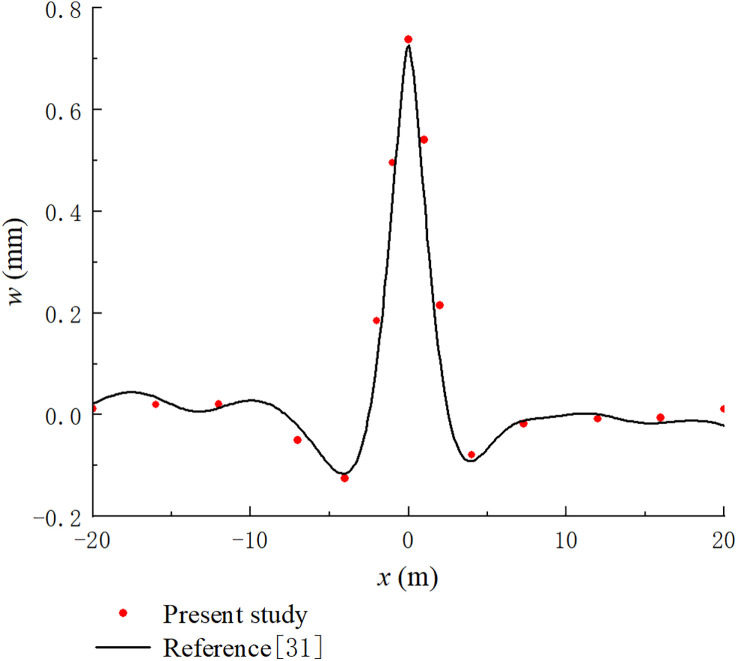
Comparison of calculated results for deflection of infinite elastic plate along the x-axis direction.

To verify the correctness of the theoretical derivations and numerical calculation results in this paper, the orthotropic anisotropic layered foundation is simplified as a single-layer homogeneous model. The case study presented in Reference [31] is adopted, and a comparative analysis is conducted with its numerical results for the plate displacement along the *x*-direction under the conditions $k_{\text{1}}=1.2$ and $k_{\text{2}}=0.8$. As can be seen in Fig 2, the numerical results obtained in this study exhibit fundamental agreement with those of Reference [31], which verifies the correctness of the theoretical derivations and computational methodology in this paper.

### 4.3. Comparison between analytical solutions and finite element analysis

#### 4.3.1. Finite element modeling.

**Fig 3:**
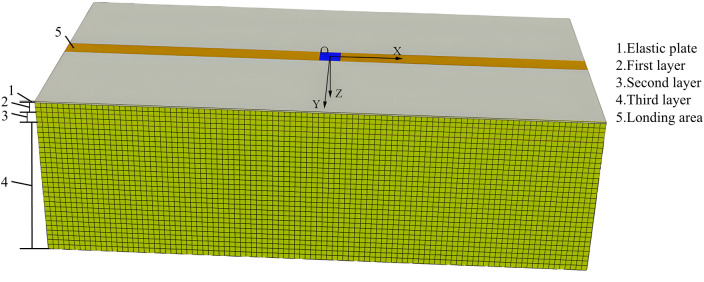
Finite element model for orthotropic soils.

Based on the ABAQUS software, a three-dimensional finite element analysis model of an infinite elastic plate overlying an orthotropic anisotropic layered foundation is established. When the thickness of the foundation soil reaches a sufficiently large magnitude, the soil can be regarded as a semi-infinite structure [38]. Considering actual engineering conditions and integrating factors such as model boundary effects and computational efficiency, the foundation model dimensions are set to 100m×50m×30m, as shown in Fig 3. In the synergistic interaction between the layered foundation and the elastic plate, vertical displacements are continuous, and frictional effects are neglected.

The foundation soil is selected as an elastic orthotropic anisotropic constitutive model, while the road plate is modeled as an elastic plate. The C3D8 solid element type is employed to discretize the foundation soil and the elastic plate into a mesh, resulting in a total of 150,000 elements. The boundary conditions are imposed as follows: normal displacement constraints are applied around the periphery of both the foundation and the plate; a fully fixed three-dimensional constraint (restricting transverse displacement of U1, longitudinal displacement of U2, and vertical displacement of U3) is implemented at the bottom of the foundation. To minimize the influence of model geometric boundaries and constraints on the dynamic response simulation results, the initial position of the moving load is positioned at the geometric center of the model.

The surface of the foundation is subjected to a moving rectangular harmonic load. Considering the complexity of the load, a DLOAD subroutine is developed using the FORTRAN programming language to implement the moving harmonic rectangular load in ABAQUS software. This subroutine controls the load moving speed, amplitude, spatial distribution, and vibration frequency, ensuring that the load moves uniformly along the positive *x*-direction.

#### 4.3.2. Calculated results.

To validate the accuracy of the finite element model, Case 4 from Section 3.1 is adopted for comparison. The numerical results are compared with the simulation results generated by the finite element software ABAQUS.

**Fig 4:**
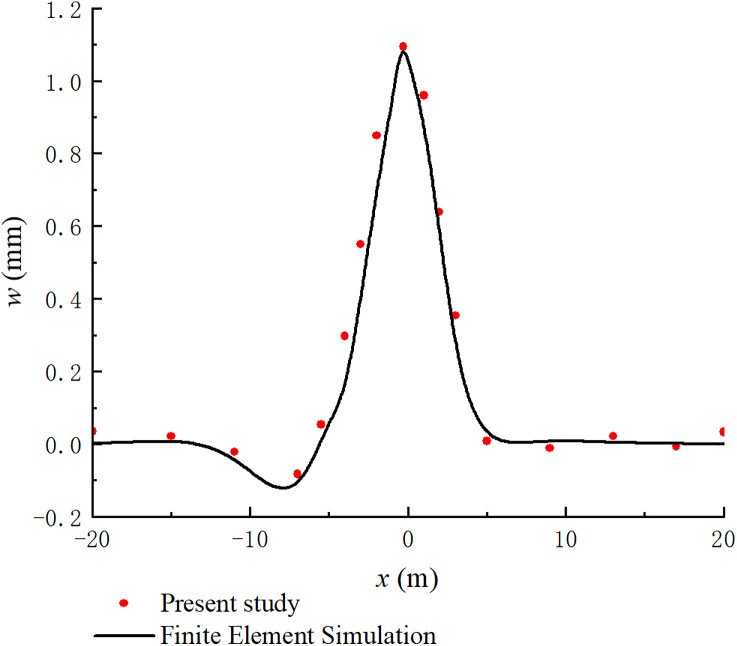
Comparison of calculated results of analytical solution and finite element simulation.

As shown in Fig 4, favorable agreement is observed between the numerical solution and the simulation analysis results from the finite element software ABAQUS. which verifies the accuracy of the three-dimensional finite element model developed in this paper.

### 4.4. Influence of the stratified foundations on plate deflection

To investigate the influence of foundation stratification characteristics on plate deflection, the differences in the dynamic response of the plate caused by the changes in the foundation parameters of each layer under seven different cases in Section 4.1 are compared, and the influence rules are analyzed.

**Fig 5:**
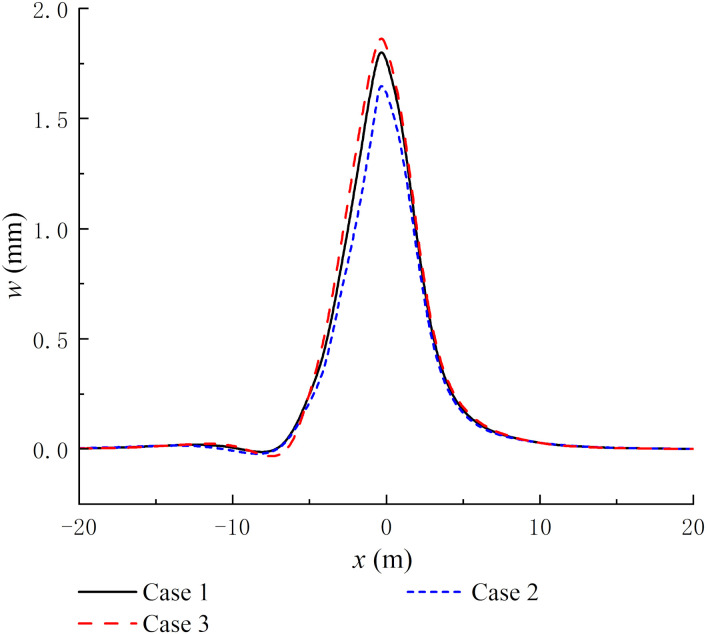
Plate deflection curves for different soil parameters of the 2nd and 3rd layers.

**Fig 6:**
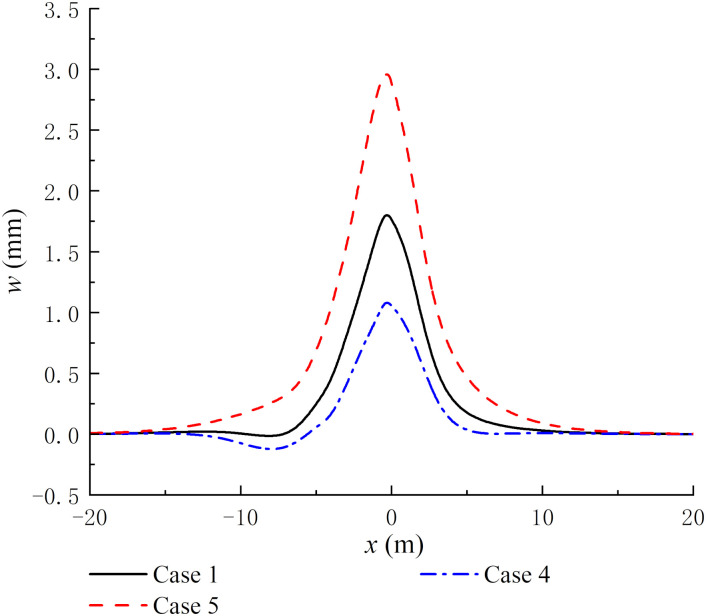
Plate deflection curves for different soil parameters of the 1st and 3rd layers.

**Fig 7:**
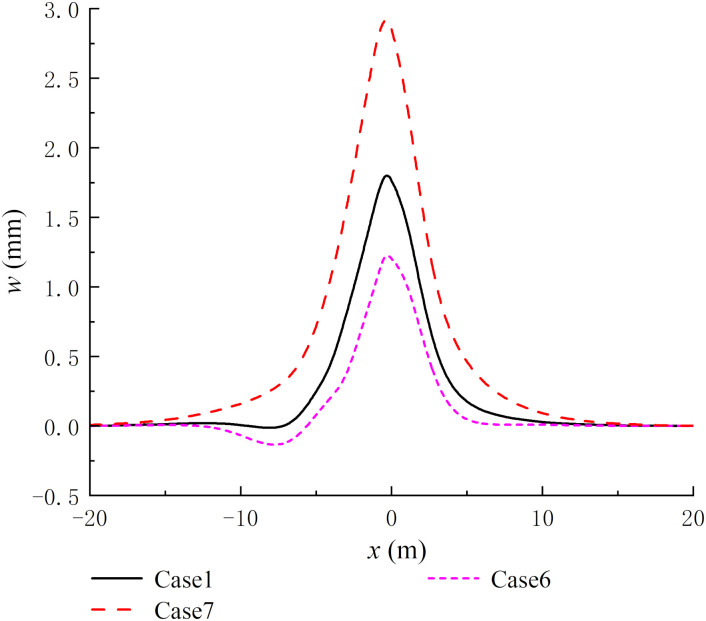
Plate deflection curves for different soil parameters of the 1st and 2nd layers.

Figs 5–7 present the distribution curves of the plate deflection response when changing the parameters of the lower-layer and upper-layer soils, respectively. Figs 5–7 demonstrate that the deflection curves of the plate mainly vary within the region of -15m to 15m, and the curve values beyond the region approach zero. Under the seven cases, the dynamic response of the plate deflection in the load center area reaches its peak value, and its influence range is basically the same. In the area beyond twice the distance of the load range, the plate deflection values rapidly attenuate and approach zero. Furthermore, the fluctuation amplitude of the deflection curve is significantly greater in the left-side region relative to the right-side region. Based on the peak deflection of Case 1 (1.80 mm) as the benchmark, Fig 5 reveals that the peak deflection of Case 2 decreases by 8.33%, while that of Case 3 increases by 3.33%. Fig 6 indicates that the peak deflection of Case 4 decreases by 40.00%, and the peak deflection of Case 5 increases by 64.44%. Fig 7 demonstrates that the peak deflection of Case 6 is reduced by 32.22%, and the peak deflection of Case 7 is increased by 62.22%. The above analysis demonstrates that the stratification characteristics of the foundation exert a significant influence on the plate deflection. Variations in the elasticity modulus and shear modulus of the upper-layer soil are observed to exert a more pronounced influence on the deflection magnitude than those of the lower-layer soil. The larger the elastic modulus and shear modulus of the upper soil, the smaller the plate deflection. Consequently, it is unjustified to model the foundation as a single-layer homogeneous medium while neglecting its inherent stratification characteristics in computational analyses.

### 4.5. Influence of orthogonal anisotropy parameters of foundations on plate deflection

**Fig 8:**
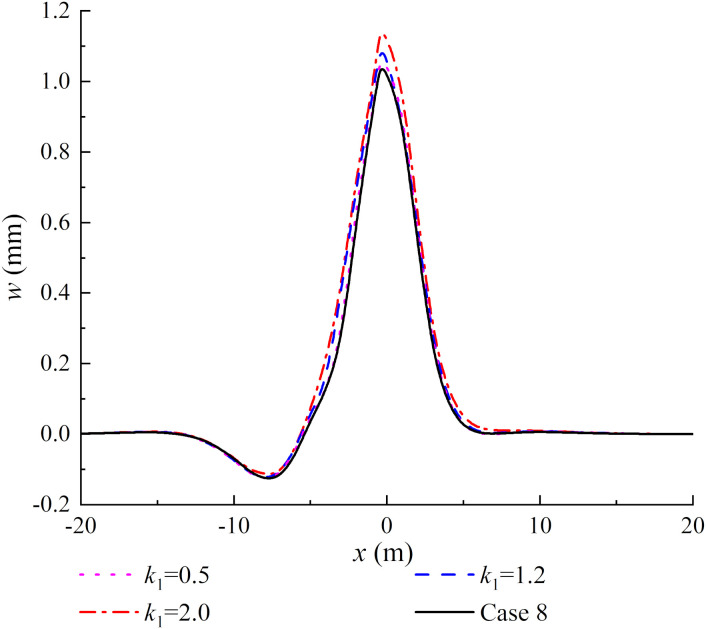
Plate deflection curves for different $E_{y}$ of the first soil layer.

**Fig 9:**
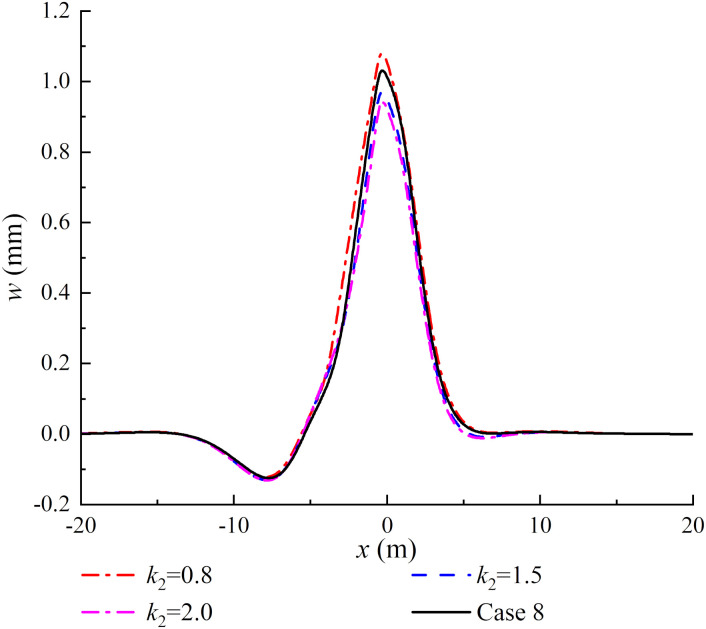
Plate deflection curves for different $E_{z}$ of the first soil layer.

The influence law of the orthotropic anisotropic parameters of the foundation on plate deflection is investigated by varying the elasticity modulus components $E_{x1}$, $E_{y1}$, and $E_{z1}$ of the first-layer soil. The parameters of the first-layer soil are specified as $E_{x1}=100\text{MPa}$, $G_{yz1}=40\text{MPa}$, $\mu_{xy1}=0.25$, and $\rho_{\text{1}}=1800kg/m^{\text{3}}$. The parameters of the 2nd and 3rd soil layers are the same as those defined in Case 4. The elasticity modulus components are defined by the ratios $E_{y1}=k_{\text{1}}E_{x1}$ and $E_{z1}=k_{\text{2}}E_{x1}$. Variations in $E_{y1}$ and $E_{z1}$ of the first-layer soil are implemented by adjusting the coefficients $k_{\text{1}}$ and $k_{\text{2}}$, with $k_{\text{1}}=1.2$ and $k_{\text{2}}=0.8$ set as default values unless otherwise specified. Case 8 is incorporated into the analysis, in which the first-layer soil is modeled as an isotropic material characterized by the parameters $E_{x1}=100\text{MPa}$, $\mu_{\text{1}}=0.25$, and $\rho_{\text{1}}=1800kg/m^{\text{3}}$, and the parameters for the second and third soil layers are maintained consistent with those defined in Case 4. As can be seen from Fig 8, under the condition of maintaining $k_{\text{2}}=0.8$, the maximum deflection of the plate continuously increases with the increase in $k_{\text{1}}$ (i.e., $E_{y1}$). An increase of 7.62% in the peak plate deflection is observed when $E_{y1}$ is increased from 50MPa to 200MPa. Compared to the curves at different $k_{\text{1}}$ values, the plate deflection amplitude exhibits the lowest magnitude when the first-layer soil is isotropic. Fig 9 reveals that, under the condition of $k_{\text{1}}=1.2$, the amplitude of the plate deflection exhibits a monotonically decreasing trend with increasing $k_{\text{2}}$ (i.e., $E_{z1}$) when only $k_{\text{2}}$ is adjusted. The maximum deflection increases by 14.89% when $E_{z1}$ is reduced from 200MPa to 80MPa. Notably, the deflection amplitude for Case 8 is second only to that of the $k_{\text{2}}=0.8$, reaching 1.03 mm. The above analysis demonstrates that the orthotropic anisotropy of the first soil layer exerts a more pronounced influence on the plate deflection relative to isotropy.

### 4.6. Influence of load parameters on plate deflection

(1) Load moving speed

**Fig 10:**
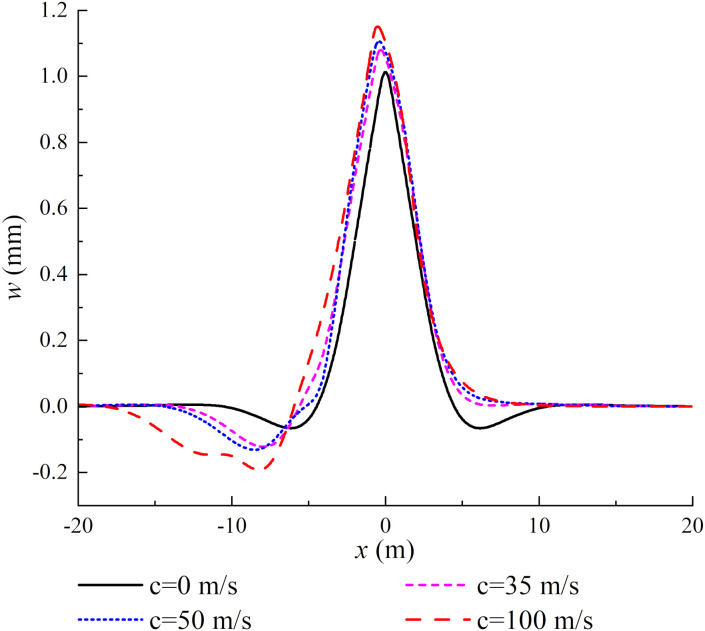
Curves of vertical displacement of soil surface for different load movement rates.

To investigate the influence of load moving speed on plate deflection within the subsonic range, four representative cases of load moving speed are selected for comparative analysis: c=0m/s (static load), 35m/s, 50m/s, and 100m/s. The computational model and example parameters are adopted from Case 4 in Section 4.1. Fig 10 presents the variation curves of plate deflection under different load moving speeds. As observed in Fig 10, the curve is symmetric about the zero point only when the speed is zero. The deflection *w* increases with higher load moving speeds, and the fluctuation and hysteresis of the curve are also significantly enhanced. When the speed increases from 0 m/s to 100 m/s, the maximum deflection of the plate increases by 13.86%.

(2) Load vibration frequency

**Fig 11:**
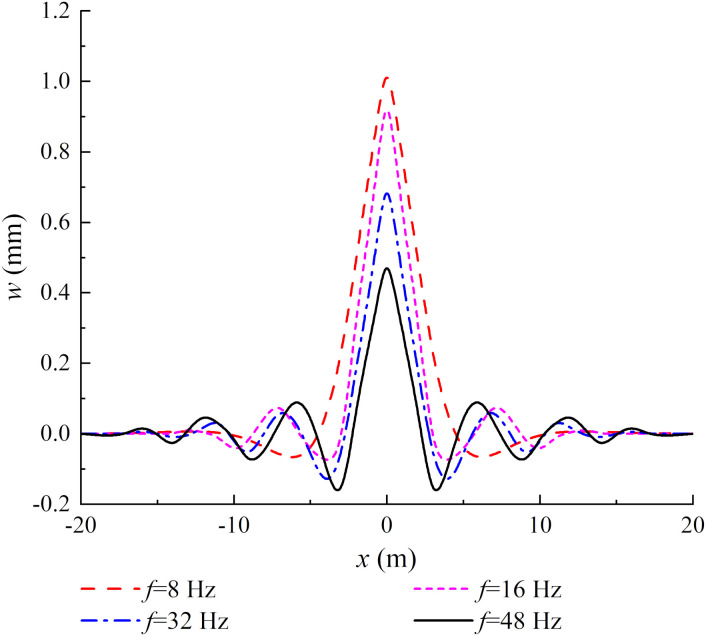
Curves of plate deflection for different loading frequencies.

The influence of load vibration frequency on plate deflection is investigated. At a load speed of zero, four distinct load vibration frequencies are selected for comparative analysis: f=8Hz, 16 Hz, 32 Hz, and 48 Hz. The remaining parameters are the same as those in Case 4 of Section 4.1. Fig 11 presents the plate deflection curves under the four load vibration frequencies. As observed in Fig 11, under a speed of zero, all deflection curves exhibit symmetry about the load application center point, with the peak amplitude consistently attained at this location. The maximum plate deflection decreases with increasing load vibration frequency. A reduction of 53.47% in the maximum deflection is achieved when the load frequency is increased from 8 Hz to 48 Hz. Concurrently, both the fluctuation and maximum negative value of the deflection curve increase with higher load frequencies.

### 4.7. Influence of elastic plate parameters on plate deflection

The influence of different plate elastic moduli and plate thicknesses on plate deflection is investigated with the computational model and example parameters taken from Case 4 in Section 4.1.

**Fig 12:**
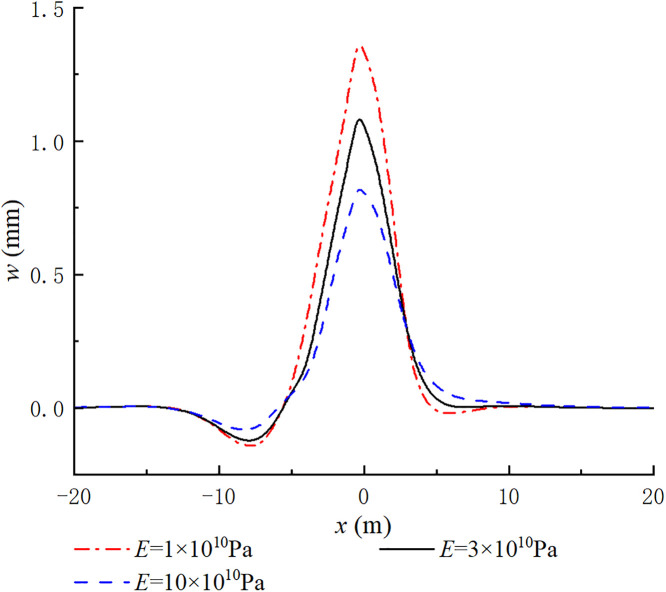
Curves of plate deflection with different plate elastic moduli.

**Fig 13:**
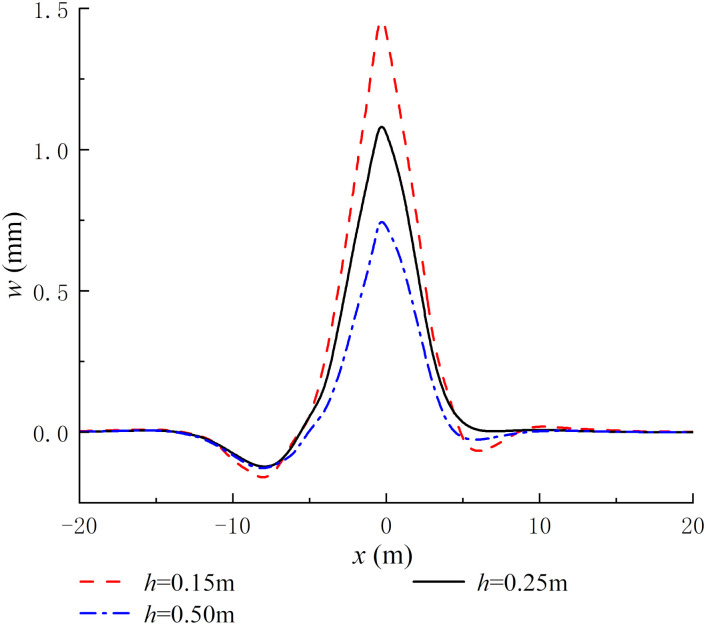
Curves of plate deflection with different plate thicknesses.

Fig 12 displays the plate deflection variation curves with different plate elastic moduli, while Fig 13 presents the corresponding curves for different plate thicknesses. As observed from Fig 12, the maximum plate deflection exhibits a decreasing trend with increasing elasticity modulus. The maximum plate deflection decreases by 40.91% when the elasticity modulus *E* is increased from $1\times 10^{10}\text{Pa}$ to $10\times 10^{10}\text{Pa}$. Fig 13 demonstrates that the amplitude of plate deflection continuously increases with decreasing plate thickness. An increase of 97.29% in the maximum plate deflection is observed when the plate thickness *h* is reduced from 0.50 m to 0.15 m. The above analysis indicates that plate deformation can be significantly reduced by increasing either the plate elasticity modulus or the plate thickness.

## 5. Conclusions

Based on the principles of matrix analysis and integral transforms, this study investigates the three-dimensional dynamic response of an infinite plate resting on a layered orthotropic anisotropic foundation subjected to moving loads. The dynamic response laws of the plate deflection influenced by the layered characteristics of the foundation, the orthotropic properties of the first soil layer, the load parameters and the elastic plate parameters are analyzed. The following conclusions are obtained:

(1) The elasticity modulus and shear modulus of layered soils have different influences on plate deflection. The first soil layer exhibits a more pronounced effect on reducing deformation response of the pavement plate compared to the other layers. This characteristic can be leveraged to optimize the response of layered structural systems under dynamic loads.
(2) The orthotropic anisotropic parameters of the upper-layer soil exert a considerable influence on the plate deflection response. In practical engineering, the orthotropic anisotropic properties of the foundation should be incorporated to obtain more accurate results.
(3) The moving speed (within the subsonic range) and vibration frequency of the load exert significant influences on plate deflection. The amplitude of the plate deflection curve increases with higher load moving speeds. With increasing load vibration frequency, the maximum value of the plate deflection curve exhibits a decreasing trend, while the curve fluctuation amplitude also intensifies.
(4) The elasticity modulus and thickness of the plate have considerable effects on its deformation. The amplitude of the plate deflection curve decreases monotonically with increasing elasticity modulus or thickness. This characteristic can be leveraged in practical engineering through structural design strategies to mitigate plate deformation.
